# Who Shoots Better: Are Left-Handers at an Advantage?

**DOI:** 10.3390/jfmk10020128

**Published:** 2025-04-10

**Authors:** Antonela Karmen Ivišić, Nikola Foretić, Dario Vrdoljak, Miodrag Spasić

**Affiliations:** 1 Faculty of Kinesiology, University of Split, 21000 Split, Croatia; antivi@kifst.hr (A.K.I.); miodrag.spasic@kifst.eu (M.S.); dariovrdoljak42@gmail.com (D.V.); 2 High Performance Sport Center, Croatian Olympic Committee, 10000 Zagreb, Croatia

**Keywords:** handball, velocity, distance, local positioning system, Kinexon, elite players

## Abstract

**Background**: Handedness dominance can be observed in the tactical aspects of a handball match geometry. Therefore, this study aimed to examine the asymmetry between shooting velocity and distance in left- and right-handed handball players, and also to see if there is a difference between scored and missed shots. **Methods**: The data were obtained from players participating in the EHF European Championship 2024, held in Germany. **Results**: In this study, 238 players were analyzed during the whole championship. They were divided into two groups: left- (N = 112) and right-handed players (N = 126). A total of 5710 shots taken by the players were collected and analyzed. The results show that the left-handed players had a higher score percentage (63.08%) than the right-handed players (57.86%). The right-handed players shot at a higher velocity (101.38 ± 18.00 km/h) than the left-handed players (99.36 ± 18.89 km/h) (*p* < 0.001). A similar difference was observed in the distance of the shots (7.61 ± 2.23 m; and 7.42 ± 2.59 m, respectively) (*p* < 0.001). The distance of the shots differed between the scored and missed shots (right-handed, *p* < 0.001; left-handed, *p* < 0.04). **Conclusions**: These findings suggest that an asymmetry in left- and right-handed players is present for both parameters. Also, the higher efficiency of the right side of a handball team could lead to asymmetry in the geometry of a handball match.

## 1. Introduction

Handball is a physically, technically, and tactically demanding team sport game [1]. Players’ performance depends on a variety of individual abilities, skills, and interactions among the different players on a team [2]. Previous studies have identified throwing velocity and precision as among the most important factors in scoring goals [3,4]. Additionally, shooting is divided into two groups: shots from the ground and jump shots. According to Rousanoglou et al. [5], a jump shot is the more common shot in handball and is physically and technically more demanding than a ground shot [5]. Regardless of the shot type, the shooting velocity is considered the most important facet of shooting efficiency [6,7].

As described in previous studies, the shooting velocity is mainly based on the anthropometric indices of the players (e.g., body mass, body height, hand size, and arm span) [8,9]. Apart from that, the playing position or distance from the goal can also be crucial for performing a precise and fast throw [10]. Most studies examining shooting by handball players have explored the biomechanical aspects of throwing motions, specifically the pattern of the optimal throwing motion, the transfer of momentum between the lower and upper body, body segment movement, and rotations [6,8,11,12]. Regarding all the above-mentioned factors that influence shooting, different playing positions have different shooting outcomes in different situations [13,14,15].

Additionally, about 10–12% of the general population is left-handed, with a disproportionately higher frequency among those playing interactive sports [16]. Equally important, handedness has gained popularity as an interesting factor among the sports population in the last two decades [17]. While there are many hypotheses that have aimed to explain the dominance of right-handedness in the human population [18,19,20], some authors have focused their attention on examining the prevalence of left-handedness in sports. Considering different sports’ demands, previous researchers have concentrated on questions of advantage. For instance, a previous study examined whether left-handedness is an intrinsic advantage in three sports: cricket, tennis, and football [21]. Others have explored whether the sport-specific ability to perceive left-hand patterns of movement is less developed when compared to right-hand patterns among tennis players [22]. In addition, Ziyagil et al. [23] examined the rate of left-handedness among top wrestlers at the world championships and showed a higher rate of left-handedness among the wrestlers with medals, for both men and women. Finally, previous authors have examined the anthropometric profiles and physical performance of elite handball players and compared left- and right-handed players [24]. Considering the interactive aspects of handball matches, it is obvious from previous studies that handedness should be considered as a potential factor. Therefore, the interesting trend of left-handed dominance in handball needs to be further analyzed.

In handball, seven players compete for each team. Games are divided into two halves of 30 min each. The main aim is to score more goals than one’s opponent [25]. The playing positions are divided into a goalkeeper, wings, backs, and pivot. Additionally, the game geometry or shooting lines could be seen as valuable for an examination of shooting. Modern handball tends to position left-handed players on the right side of the offense and the left side of the defense due to their advantages in those playing positions. Moreover, coaches often place left-handed players as center backs because of their different movement patterns, which gives them a tactical advantage. According to Rivilla-Garcia, Calvo, and Van den Tillaar [10], second-line players shoot at a higher velocity than first-line players. The authors concluded that the demands of the playing lines and anthropometry define the differences between the offensive lines. On the other hand, Zapartidis et al. [26] found no differences in the throwing velocity among young female handball players based on their playing positions. However, shooting efficiency in handball is determined through the percentage of shots scored.

The shooting velocity represents a crucial factor with a major influence on handball game outcomes [2]. A higher shooting velocity decreases the reaction time for goalkeepers to assess the ball’s position. Moreover, a higher shooting velocity exerts immense pressure on the defenders. Likewise, an assessment of shooting velocity can provide key information to help coaches, players, and experts understand performance factors [27]. Because it is difficult to measure ball velocity directly during a match, previous studies have mostly used radar guns, photocell gates, or lab-made laser devices [28,29,30]. However, automatic position detection provides insight into a player’s tracking variables during a match [31]. Therefore, the use of a local positioning system (LPS) is needed to monitor players’ shooting velocity when it comes to elite team sports [32].

From the above discussion, it is clear that different factors influence shooting in handball. Apart from that, this brief literature review shows sparse evidence from comparisons of left- and right-handed players’ shooting. Therefore, the aim of this study is to use an LPS to examine the asymmetry between shooting velocity and distance in left- and right-handed handball players, as well as to see whether there is a difference between the shots that are scored and those that are missed.

## 2. Materials and Methods

### 2.1. Participants

The sample of participants included 238 players who were analyzed during the whole championship. From these players during 65 matches, 5710 shots were collected and analyzed. This study was carried out during the EHF European Championship 2024, held in Germany. The participants were divided into two groups: left- (N = 112) and right-handed players (N = 126)). Furthermore, the inclusion criteria were as follows: players playing at left-wing, left-back, right-wing, and right-back positions; and players who were left- and right-handed. The exclusion criteria were as follows: players who played at center-back, pivot, and goalkeeper positions (N = 214); and players who did not play and hence did not have any obtainable shots for this analysis. With this, we managed to maintain an equal number of left- and right-handed players.

Additionally, all players wore official Kinexon equipment in accordance with competition regulations. Experimental procedures were completed following the Declaration of Helsinki, and they were approved by the corresponding authors’ institutional research ethics board (Ethics Board Approval No. 2181-205-02-05-25-0026).

### 2.2. Procedure

Data collection took place during the EHF European Championship 2024. The competition was held throughout January in Germany. During all stages of the competition (preliminary, main, and knockout) data were gathered succeeding each match. The players were involved in a total of 65 matches. All data were collected with a Kinexon LPS system through iBall and player tracking. The data were processed on the spot and then obtained from an online Kinexon server. One researcher obtained online available data for further analysis.

### 2.3. Variables

The variables included anthropometric indices and the velocities and distances of the shots.

**Figure 1:**
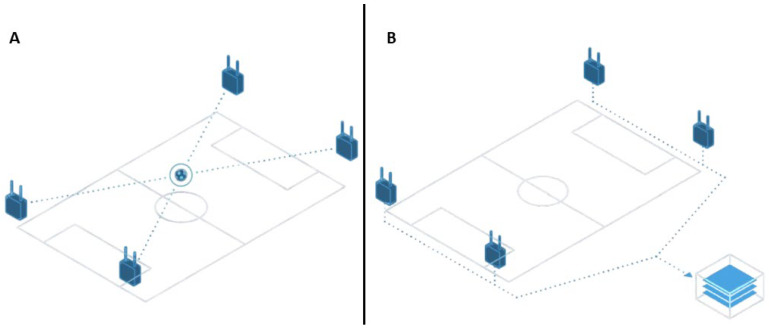
LPS and iBall Kinexon system. (**A**) Panel shows the antennas that gathered a signal from the iBall; (**B**) panel shows data acquisition by the processor.

The velocity and distance of the shots were derived from a tracking system developed for handball players (Kinexon: München, Germany; Select Sport. Glostrup, Denmark) (see Figure 1). The ball was tracked with an iBall system (SELECT, Denmark). Both parameters were observed for all shots, which were divided into goals and misses. An ultra-wideband (UWB) local positioning system (LPS) was used to assess specific movements in handball [32]. The system used in this study consisted of 14 antennas positioned around the handball court at three different heights. A tag was placed in the center of each player’s upper back using the manufacturer’s harness. The data were collected at 20 Hz and processed via the specific Kinexon Software (ver. 3.2.6, Kinexon Web Application, Munich, Germany). The signals were transmitted to the antennas using UWB technology at a frequency range of 4.25–7.25 GHz. The field position of the tag was calculated using a proprietary algorithm based on a combination of different methods, such as Time Difference of Arrival, Two-Way Ranging, and Angle of Arrival [31]. A 12-camera Vicon motion analysis system (Vicon Nexus T40, Vicon Motion Systems, Oxford Metrics, UK) was implemented in two configurations. Data were collected at 250 Hz. Only one 14 mm reflective marker (B&L Engineering, Santa Ana, CA, USA) was placed on the Kinexon tag. The data obtained from the three-dimensional marker position were used for further analysis. The marker signal was never lost for more than 25 successive images (i.e., 0.1 s) and was automatically extrapolated with Vicon 3D software (ver. 1.8.5, Vicon Motion Systems Inc., Los Angeles, CA, USA) using the marker positions immediately before and after the loss. The average Vicon calibration errors (Image and World Error, respectively) were 0.09 and 0.17 mm for data collected in the center of the court, and 0.08 and 0.16 mm for those collected on the sides of the court. The original datasets from Kinexon were oversampled from 20 to 250 Hz for subsequent fine synchronization with the Vicon data. Signals from both systems were filtered using a 3rd-order zero-phase-shifting low-pass Butterworth filter with a 10 Hz cut-off. Each pair of Kinexon and Vicon data for each movement repetition was manually synchronized to determine a common start and end. The distance traveled was then calculated as the sum of the instantaneous positions in the horizontal plane (x, y). Velocity and acceleration data were obtained using successive derivation and low-pass filtering (10 Hz, 3rd-order zero-phase-shifting Butterworth filter). Peaks in speed, acceleration, and deceleration were calculated from the raw data and utilized for the analysis of situational power performance in top-level handball, with these parameters computed as the maximum mean speed, acceleration, and deceleration over a 500 ms window, respectively [33,34].

### 2.4. Statistical Analysis

Descriptive statistics were measured to assess the arithmetic means and standard deviations (SDs) of all measured variables. The K–S (Kolmogorov–Smirnov) test for normality was used to determine the normal distribution of the data. Furthermore, to assess differences between right- and left-handed players in distance and velocity, a *t*-test for independent samples was used. Also, an independent *t*-test was used to determine the differences between shots that were missed and those that were scored. The *p*-values for all analyses were tested with a magnitude-based Cohen’s effect size (ES) statistic with modified qualitative descriptors (trivial, ES < 0.2; small, ES = 0.21–0.60; moderate, ES = 0.61–1.20; large, ES = 1.21–1.99; and extremely large, ES > 2.0). Lastly, to determine the relation between distance and velocity, Pearson’s test was used.

All analyses were performed in the statistical package Statistica ver. 13.5 (Tibco Inc., Palo Alto, CA, USA), with a p-level of 0.05 applied.

## 3. Results

The participants’ chronological age was 27.11 ± 4.47 years, with a body height of 190.95 ± 6.67 cm, and body mass of 91.18 ± 9.36 kg, determined from the official statistical data provided by the EHF. The anthropometric indices showed that the right-handed players were heavier (92.38 ± 9.69 kg) and taller (192.22 ± 6.99 cm) than the left-handed players (89.83 ± 8.83 kg and 189.53 ± 6.01 cm, respectively).

**Figure 2:**
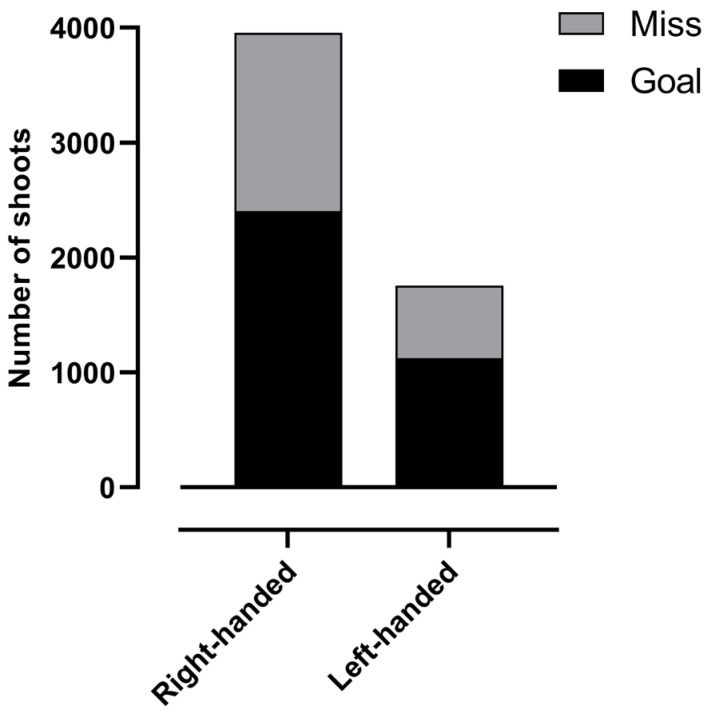
Number of goals and misses for right- and left-handed players.

Figure 2 presents the percentage of goals and misses of the shots, according to playing positions and right-/left-handed players. The results show that the left-handed players had a higher score percentage (63.08%) than the right-handed players (57.86%). However, the right-handed players shot more often (3955) and scored more goals (2385) than the left-handed players (1755 and 1107, respectively).

**Figure 3:**
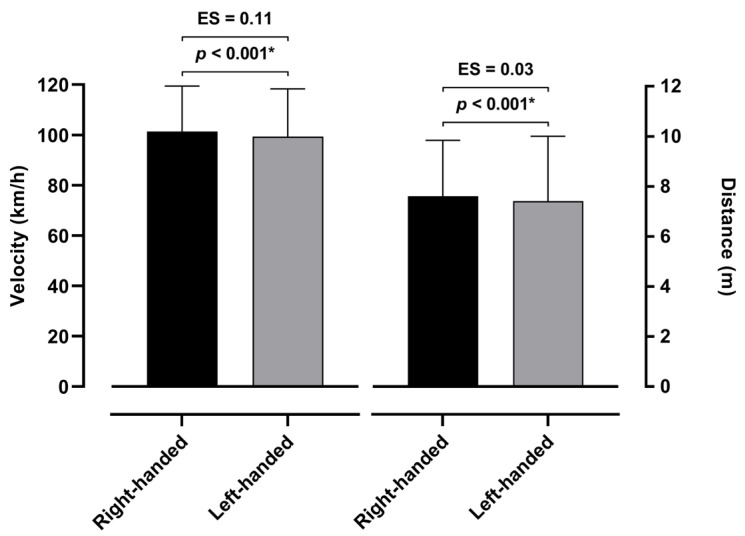
Differences between left- and right-handed players in velocity and distance of shots, with *p*-level < 0.05 marked with *.

The differences in the velocities and distances of the shots between the right- and left-handed players can be observed in Figure 3. The analysis demonstrates a significant difference in both the velocity (*p* < 0.001) and distance (*p* < 0.001). Precisely, the right-handed players shot at a higher velocity (101.38 ± 18.00 km/h) than the left-handed players (99.36 ± 18.89 km/h). A similar difference can be observed in the distance of the shots (7.61 ± 2.23 m and 7.42 ± 2.59 m, respectively). Furthermore, the effect size analysis demonstrates small sizes between both variables, precisely, 0.11 for the velocity of the shot and 0.03 for the distance between the right- and left-handed players.

**Figure 4:**
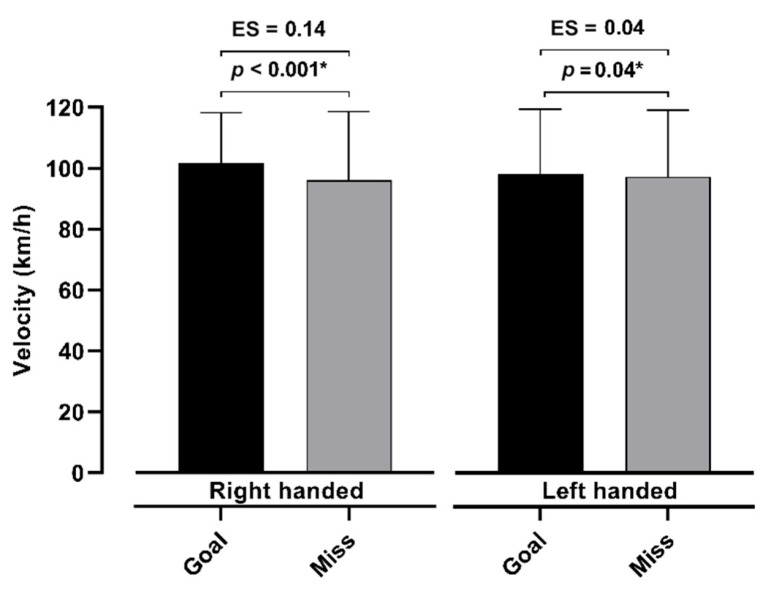
Differences between goals and misses with respect to velocity of shots for left- and right-handed players, with *p*-level < 0.05 marked with *.

**Figure 5:**
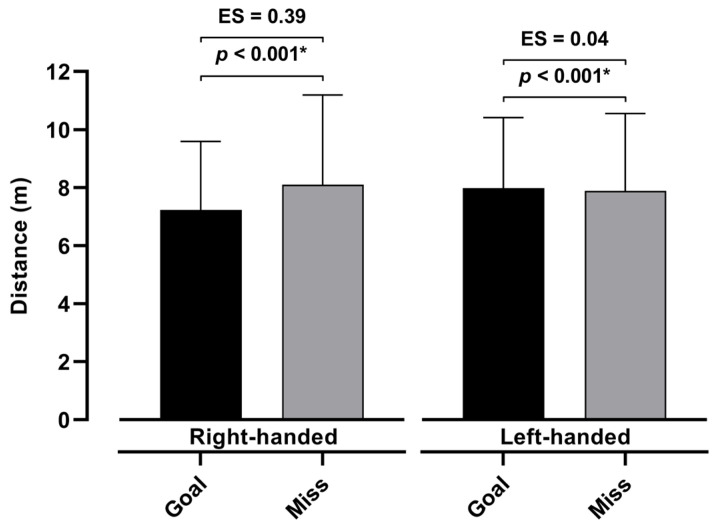
Differences between goals and misses with respect to distance of shots for left- and right-handed players, with *p*-level < 0.05 marked with *.

**Figure 6:**
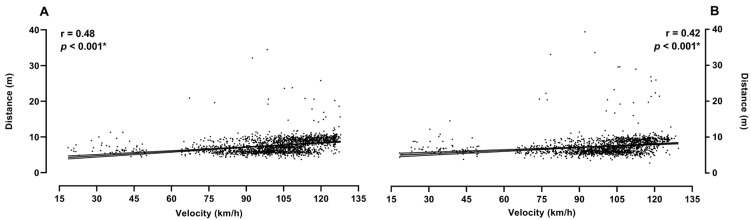
Correlation analysis between velocity and distance for both right- (**A**) and left-handed (**B**) players; with * showing significance at *p*-level < 0.05.

Figure 4 and Figure 5 show the differences between the goals and misses with respect to the velocity and distance of the shots for both the right- and left-handed players. The results show that the velocity of the shots that were goals was significantly higher than those that were misses for the right- (*p* < 0.001) and left-handed players (*p* = 0.04). Similarly, the distance of the shots differed between the scored and missed shots (right-handed, *p* < 0.001; left-handed, *p* < 0.001). Additionally, the effect sizes demonstrate the small effect of the sample on both the distance and velocity when goals and misses are compared. Also, Figure 6 demonstrates the correlations between the velocity and distance of the shots for both the left- and right-handed players. The results show a statistically significant correlation of 0.48 (*p* > 0.001) for the right-handed and 0.42 (*p* > 0.001) for the left-handed players.

## 4. Discussion

Although the main aim of this study was to analyze the differences between left-handed and right-handed players, the results indicate a few important findings: (i) left-handed players have a higher scoring percentage; (ii) right-handed players shoot from longer distances at greater velocities; and (iii) both groups shoot at greater velocities at larger distances.

According to the present analysis of the scoring percentages, the results show that the left-handed players scored more than the right-handed players. Some authors have previously highlighted an unusually high proportion of left-handed players among top-level athletes [17]. Others have proposed that the advantages of left-handed athletes are mostly due to differences in certain lateralized processes (e.g., spatial orientation and attention and cued recall in the right cerebral hemisphere, which controls the left hand) [35]. Moreover, Dane and Erzurumluoglu [36] suggested that left-handed handball players have an intrinsic neurological advantage in visual reaction-time tests. Consequently, when it comes to the tactical and strategic components of handball matches, left-handed players may have an advantage in unfamiliar situations. In other words, defense players are mostly familiar with right-handed players due to right-hand dominance in general. Conversely, left-handed players do not need to reverse their usual strategy when playing an offensive part in a match. One of the typical tactical uses of this advantage by handball coaches is choosing left-handed players for 7 m shooting. Most of the time, these shots are performed by right-wing players who shoot with the left hand and have a combination of unusual movement patterns and very efficient and precise handball techniques. These characteristics make their shots more demanding for goalkeepers.

A longer distance and greater shooting velocity were noticed in the right-handed players’ results. This finding could be interpreted as being due to the anthropometric indices of the athletes. As previously mentioned, the right-handed players had a higher body mass when compared to the left-handed players. Hence, their greater body mass may be the reason for their greater shooting velocity. Likewise, previous studies have demonstrated that body mass appears to have the highest influence on throwing velocity among handball players [9]. Furthermore, heavier handball players have greater muscle mass [9], which is the most important factor in force and power output [37]. Defensive players generally have a greater body height, which is influential when playing an offensive part at large distances. According to Hatzimanouil [38], a lower body height influences shots made at shorter distances. Additionally, previous studies have reported that the body height of elite-level handball back players is 192.6 and 193 cm [39,40]. Consequentially, shorter players run more rapidly during transitional attacks. Furthermore, most of the shots that they perform are taken without making significant contact, exploiting their speed and agility [41]. On the other hand, taller players tend to shoot from longer distances due to their playing positions and match demands [41].

Finally, the measured shooting velocity and effectiveness (score) indicate that both the left- and right-handed players scored with greater velocities at greater distances. Similar to our results, previous studies have shown that significantly more goals are scored from distances of less than 9 m [38]. Such findings indicate that fast and rapid breaks through defensive lines increase the possibility of scoring. Moreover, the velocity of the shots differed significantly between goals and misses. However, the observation of the effect sizes showed that such differences had little effect. This can be explained by the playing positions. Both the left- and right-handed players played in similar positions (i.e., wings and backs). Therefore, their mean velocities were similar.

The effect size analysis of the significance of the previously mentioned factors, as well as the goals and misses between the left- and right-handed players, showed low values. Such findings imply low practical applications of the observed differences. Specifically, the right-handed players shot with 2.02 km/h greater velocity. When it comes to the training load, this velocity increase does not contribute to performance efficiency. Additionally, this difference does not have practical implications for training regimes and probably will not be a determinant of shooting efficacy in handball matches.

Furthermore, the distance difference was only 0.19 m in favor of the right-handed players. Similarly to the velocity results, a difference as small as 0.19 m is not significant in either the tactical or strategic part of handball training. Presumably, the presented distance is not of pivotal importance for the efficiency of a handball game, so it does not need to be considered in the domain of practical application to the training workload. Yet, the lack of practical applications of this study’s findings has led us to consider further studies to examine the importance of dexterity in handball. Those studies should analyze dexterity differences in the context of playing positions and game situations. The resulting data could provide deeper insight into the abovementioned issues and give more practical information about the training load, tactical aspects, and role selection in handball.

## 5. Conclusions

As previously mentioned, handedness dominance in sports is seen as an interesting field of research. This trend can be observed in the tactical aspect of a handball match geometry. Therefore, this study aimed to examine the asymmetry between shooting velocity and distance in left- and right-handed handball players by using an LPS, as well as to see whether there is a difference between the shots that are scored and those that are missed. The results show that left-handed players have higher shooting efficiency. Furthermore, right-handed players shoot from longer distances with greater velocities. Additionally, goals are scored at lower velocities and smaller distances. However, the effect sizes of the differences are small.

To conclude, these findings suggest that asymmetry is present in both parameters between left- and right-handed players. In addition, the higher efficiency of the left side of a handball team can lead to asymmetry in the geometry of handball matches. The findings of this study could assist handball coaches in gaining a deeper understanding of the tactical nature of the game. Further analysis is needed to unravel the intriguing question of why most attacks finish on the left side, despite left-handed players demonstrating superior shooting efficiency. Future research should explore this phenomenon, considering factors such as player skill levels and overall team quality.

## Data Availability

All data are presented in the manuscript.

## Abbreviations

The following abbreviations are used in this manuscript:

LPS	Local positioning system
EHF	European handball federation
